# Post-Infectious Myocardial Infarction: New Insights for Improved Screening

**DOI:** 10.3390/jcm8060827

**Published:** 2019-06-11

**Authors:** Alain Putot, Frédéric Chague, Patrick Manckoundia, Yves Cottin, Marianne Zeller

**Affiliations:** 1 Geriatrics Internal Medicine Department, Dijon University Hospital, 21079 Dijon CEDEX, France; patrick.manckoundia@chu-dijon.fr; 2 Physiopathologie et Epidémiologie Cérébro-Cardiovasculaires (PEC2), EA7460, Université de Bourgogne Franche-Comté, 21078 Dijon CEDEX, France; frederic.chague@chu-dijon.fr (F.C.); yves.cottin@chu-dijon.fr (Y.C.); marianne.zeller@chu-dijon.fr (M.Z.); 3 Cardiology Department, Dijon University Hospital, 21079 Dijon CEDEX, France

**Keywords:** infection, Type 2 myocardial infarction, in-hospital mortality, sepsis, acute coronary syndrome, pulmonary tract infection, pneumonia, elderly

## Abstract

Acute infection is suspected of involvement in the onset of acute myocardial infarction (MI). We aimed to assess the incidence, pathogenesis and prognosis of post-infectious MI. All consecutive patients hospitalized for an acute MI in coronary care units were prospectively included. Post-infectious MI was defined by a concurrent diagnosis of acute infection at admission. Type 1 MI (acute plaque disruption) or Type 2 MI (imbalance in oxygen supply/demand) were adjudicated according to the universal definition of MI. From the 4573 patients admitted for acute MI, 466 (10%) had a concurrent acute infection (median age 78 (66–85) y, 60% male), of whom 313 (67%) had a respiratory tract infection. Type 2 MI was identified in 72% of post-infectious MI. Compared with other MI, post-infectious MI had a worse in-hospital outcome (11 vs. 6% mortality, *p* < 0.01), mostly from cardiovascular causes. After adjusting for confounders, acute infections were no more associated with mortality (odds ratio 0.72; 95% confidence interval 0.43–1.20). In the group of post-infectious MI, Type 1 MI and respiratory tract infection were associated with a worse prognosis (respective odds ratio 2.44; 95% confidence interval: 1.12–5.29, and 2.89; 1.19–6.99). In this large MI survey, post-infectious MI was common, accounting for 10% of all MI, and doubled in-hospital mortality. Respiratory tract infection and Type 1 post-infectious MI were associated with a worse prognosis.

## 1. Background

Acute infections are known to be associated with an increased risk of myocardial infarction (MI), especially respiratory tract infection, including pneumonia, bronchitis and influenza, but also digestive and urinary tract infections [1,2]. The most pointed evidence of this link is the flu-like seasonality of MI incidence [3] and the efficacy of influenza vaccination for MI prevention [4]. Experimental data also supports a causal relation between acute respiratory tract infection and acute coronary syndromes [1]. However, despite the identification of acute infection as a causal factor, diagnosis of post-infectious MI is not usually individualized in clinical practice, and there is a disconcerting lack of prospective observational data in cardiology units for this frequent condition.

The mechanisms underlying the triggering of post-infectious MI by acute infection could include both coronary endothelial dysfunction [5] and platelet activation, and subsequent coronary thrombosis [6], but also a sepsis-related increase in myocardial oxygen consumption leading to functional MI. Type of MI are defined according to universal MI definition [7]. Type 1 MI is the classical MI, linked to atherothrombotic coronary artery disease and usually caused by atherosclerotic plaque rupture or erosion. Type 2 MI, is an emerging pathophysiological concept corresponding to a mismatch between myocardial oxygen supply and demand, leading to ischemic myocardial injury [8]. Type 3 MI corresponds to patients with suspected acute myocardial ischemic event, but who died before cardiac biomarkers can be obtained. Type 4 and 5 are coronary revascularization-related myocardial injury (i.e., percutaneous coronary intervention or coronary artery bypass grafting), as periprocedural issues or later device-related complications. Acute infection is increasingly suspected of being a frequent cause of Type 2 MI [9,10,11]. However, the respective frequency of these two pathophysiological mechanisms in post-infectious MI remains unclear.

Numerous series have previously highlighted the increased risk of MI among outpatients [12] or inpatients [6,13,14,15], hospitalized for acute infection, but, to the best of our knowledge, the burden of this association in terms of incidence and outcome has not been studied among MI patients from cardiology units.

In this first study of post-infectious MI from a large prospective MI survey in coronary care units, we aimed to comprehensively characterize this under-recognized association and to determine factors associated with in-hospital prognosis, including the Type of MI according to the universal definition.

## 2. Methods

### 2.1. Patients

The characteristics of the French regional obseRvatoire des Infarctus de Côte d’Or (RICO) survey have been described elsewhere [16]. Briefly, RICO is an ongoing survey that prospectively collects data from patients hospitalized for MI in all coronary care units of public centers or privately funded hospitals of one eastern region of France. From October 1st, 2012 to March 31st, 2017, all consecutive patients admitted for Type 1 or Type 2 MI within 24 h after symptom onset were included in the present study, according to the third universal definition of MI [17]. Given the iatrogenic nature of Type 4 and 5 MI, and the lack of biomarkers in Type 3 MI, they were not included in the study.

The present study complied with the Declaration of Helsinki and was approved by the Ethics Committee of the Dijon University Hospital. Each patient gave written consent prior to participation.

### 2.2. Definitions

Post-infectious MI was defined as a concurrent diagnosis of acute infection at the onset of MI symptoms (i.e., acute infection symptoms preceded MI symptoms). Acute infection was defined on the basis of physician diagnosis by the presence of evocative signs or symptoms (e.g., cough, sputum, dyspnea, rhonchi or crackles for respiratory tract infection; dysuria, suprapubic or flank pain with positive urine culture or test strip for urinary tract infection) and at least one of the following clinical criteria upon admission: fever >39 °C, tachypnea >24 breaths/min, tachycardia >100 beats/min, leukocytes >12 × 10^9^ /L [18].

Acute pneumonia was diagnosed by the presence of respiratory tract infection signs or symptoms and a new infiltrate in chest imaging [19]. Acute bronchitis was diagnosed by the presence of such signs without new radiologic infiltrate.

Type of MI was defined according to the 3rd universal definition of MI [17]. Type 1 MI was defined as MI related to ischemia due to a primary coronary event such as plaque erosion or rupture, intraluminal thrombus or coronary dissection, at coronary angiography. Type 2 MI was defined by the absence of evidence of plaque rupture at coronary angiography and at least one of the prespecified supply/demand mismatch conditions, including acute infections, at the onset of MI symptoms [9,10,20].

Each case was reviewed and adjudicated by two independent reviewers (one cardiologist and one internist). Any discrepancies were resolved by consensus after an in-depth review of the patient’s medical records.

### 2.3. Data Collection

Demographic data, cardiovascular risk factors and history were collected, as were on admission ECG, clinical and biological data. The GRACE risk score was also calculated [21]. Blood samples were taken on admission to measure C-reactive protein (CRP), hemoglobin level, plasma NT-pro Brain Natriuretic Peptide and serum creatinine; estimated glomerular filtration rate was calculated using the Chronic Kidney Disease-EPIdemiology Collaboration formula (CKD-EPI). Troponin I peak was obtained from three systematic blood samples taken every 8 h in the first 24 h after admission. Troponin I tests were carried out in each hospital with conventional method (i.e., no high-sensitive assay). Dimension Vista luminescent oxygen channeling (LOCI™) troponin I assay (Siemens) [22] was used for 82% of patients. The left ventricular ejection fraction (LVEF) was measured by echocardiography in 4397 patients (96%). Coronary angiography data (4471 patients, 96%) including SYNTAX score [23] and rate of reperfusion (percutaneous coronary intervention and coronary bypass surgery) were also collected. Stenoses ≥50% at coronary angiography were considered as significant and <50% diameter stenoses were considered as non-obstructive.

### 2.4. Outcomes

In-hospital events were recorded, including all-cause and cardiovascular death (i.e., fatal MI, fatal stroke, fatal pulmonary embolism, death due to cardiogenic shock or ventricular rhythm disorders, death from cardiovascular cause, investigation or procedure. sudden unexpected death). Re-infarction was defined as occurring within 28 days after the index MI. Severe heart failure was defined by maximal Killip class 3–4 (i.e., acute pulmonary edema or cardiogenic shock).

### 2.5. Statistical Analyses

Continuous variables were expressed as mean ± standard deviation or median and interquartile ranges. A Kolmogorov-Smirnov test was performed to analyze the normality of continuous variables. The Student’s t-test or the Mann-Whitney test was used to compare continuous variables, and Chi 2 or Fisher’s tests were used to compare dichotomous data, as appropriate.

Logistic regression model was used to compare post-infectious MI patients with other MI patients, including the relevant variables associated with mortality, with a threshold at 5% in univariate analysis (age, sex, history of chronic obstructive pulmonary disease, diabetes, chronic kidney disease or heart disease, coronary artery disease, heart rate at admission, systolic blood pressure, acute heart failure, chest pain, ST segment elevation, troponin I peak value).

In the post-infectious MI group, the association between the variables of interest at admission and all-cause in-hospital mortality were assessed by logistic regression analysis using univariate analysis, and then in multivariate models including the variables associated with mortality, with a threshold at 5% in univariate analysis (GRACE score, LVEF, CRP, NT-pro Brain Natriuretic Peptide) and pre-specified relevant variables (Type 1/Type 2 MI, respiratory tract infection). Logistic regression models were also used to study the variables associated with in-hospital mortality in the entire post-infectious MI population and among patients who underwent coronary angiography. The threshold for significance was set at 5%. SPSS version 12.0.1 (IBM Inc., Armonk, NY, USA) was used for all statistical testing.

## 3. Results

### 3.1. Baseline Characteristics

**Figure 1:**
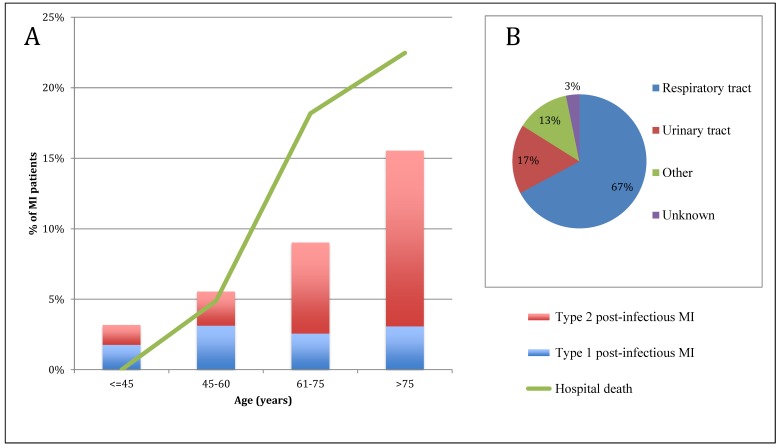
(**A**) Proportion of post-infectious myocardial infarction (MI) in patients hospitalized for MI in coronary care units, per age group and pathophysiological type of MI, and in patients dead in hospital. (**B**) Distribution of infection site in post-infectious MI.

**Figure 2:**
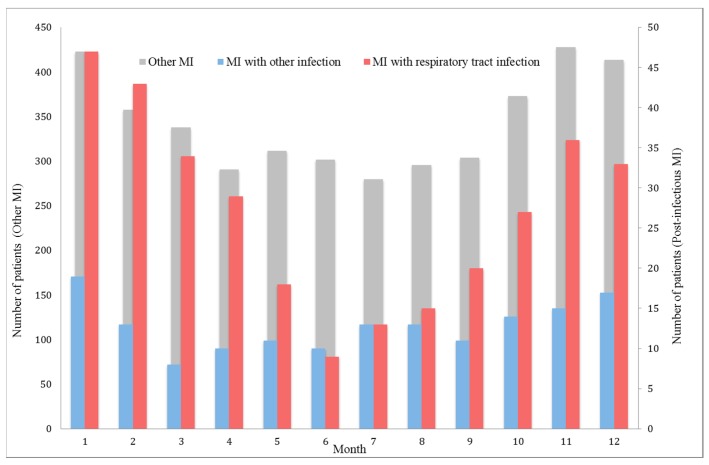
Incidence of post-infectious myocardial infarction (MI) and other MI per month of the year.

**Figure 3:**
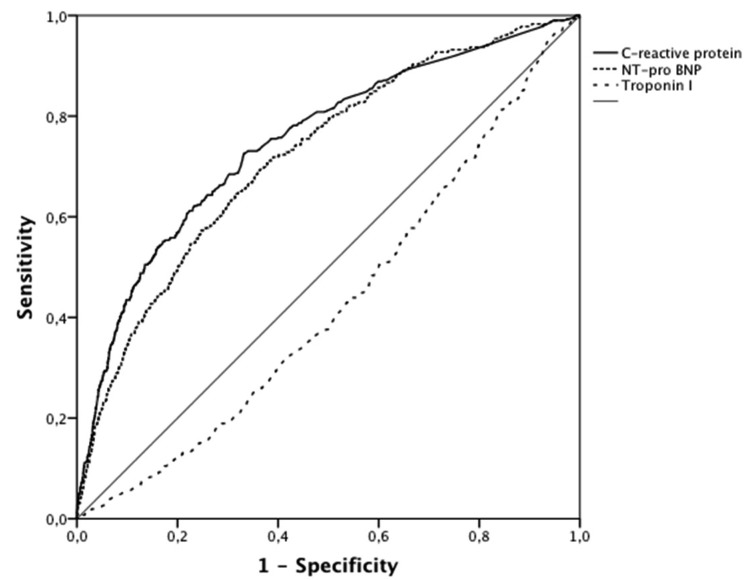
Receiver operating curves of predictive biomarkers for post-infectious myocardial infarction.

Among the 4573 patients hospitalized for acute MI, 466 (10%) had post-infectious MI. The proportion of post-infectious MI gradually increased with age (Figure 1). No significant difference in post-infectious MI frequency was observed between the different hospital settings. Seasonality of post-infectious MI and other MI is presented in Figure 2. Patient characteristics are presented in Table 1. Post-infectious MI patients were 10 years older than patients with MI without acute infection (median age 78 (66–85) vs. 68 (57–80) y, *p* < 0.001), more frequently female (40% vs. 28%, *p* < 0.001) and had more comorbidities and cardiovascular history (i.e., coronary artery disease (CAD): 36% vs. 24%, *p* < 0.001). Acute clinical presentation was more severe in post-infectious MI (i.e., acute heart failure: 54% vs. 23%, *p* < 0.001 and GRACE risk score 177 (151–199) vs. 144 (121–173), *p* < 0.001). Electrocardiogram (ECG) at admission for post-infectious MI showed less frequent ST segment elevation (39% vs. 48%, *p* < 0.001) and a higher rate of new onset or chronic atrial fibrillation or flutter (14% vs. 9%, *p* < 0.001). In addition, peak troponin I was much lower in post-infectious MI patients (7 (2–29) vs. 13 (3–58) µg/L) whereas NT-pro Brain Natriuretic Peptide level was almost 6 times higher (3800 (920–12,772) vs. 664 (169–2685) pg/mL). As expected, C-reactive protein level was much higher in the post-infectious MI group (33 (7–103) vs. 5 (3–13) mg/L). Areas under the Receiver Operating Curves for biomarkers of post-infectious MI are presented in Figure 3.

**Figure 4:**
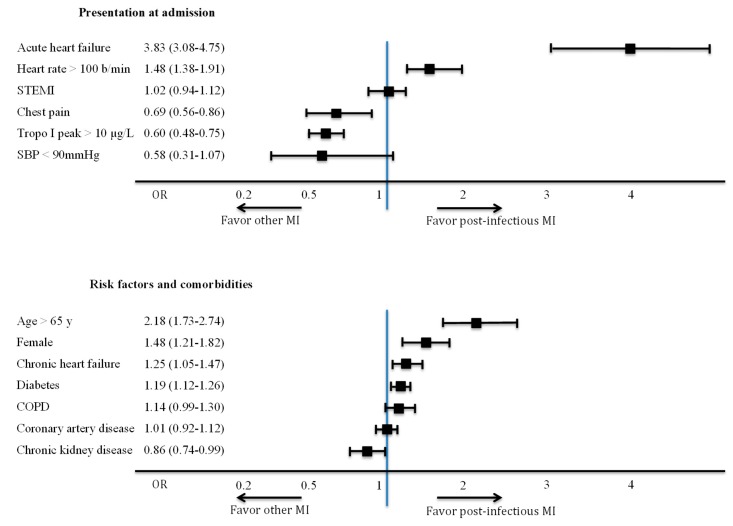
Multivariate analysis of factors associated with post-infectious myocardial infarction (Odds ratio (95% confidence interval)). COPD: chronic obstructive pulmonary disease; MI: myocardial infarction; SBP: systolic blood pressure; STEMI: ST segment elevation myocardial infarction; Tropo I peak: cardiac troponin I peak.

Key characteristics of post-infectious MI, including clinical and ECG signs of ischemia and pattern of infections are detailed in Table 2. More than half (55%) had ischemic chest pain, most (82%) had new ECG abnormalities, and only a few (13%) had imaging evidence of acute ischemia. Clinical parameters associated with post-infectious MI and subgroups at risk of post-infectious MI in multivariate analysis are presented in Figure 4. At admission, acute heart failure was strongly associated with post-infectious MI (OR (95% CI) = 3.83 (3.08–4.75)), whereas chest pain (OR (95% CI) = 0.69 (0.56–0.86)) and troponin I >10 µg/L (OR (95% CI) = 0.60 (0.48–0.75)) were associated with a lower risk of post-infectious MI. No association was found with ST segment elevation (OR (95% CI) = 1.02 (0.94–1.12)). Patients aged > 65 y, women and patients with chronic heart failure or diabetes were at higher risk of post-infectious MI than other MI.

The type of acute infections leading to post-infectious MI (Figure 1, Table 2) were mainly respiratory tract infections (*n* = 313, 67%), including acute bronchitis (*n* = 163, 35%) and acute pneumonia (*n* = 150, 32%), followed by urinary tract infections (*n* = 78, 17%), other site infections (*n* = 60, 13%) and infections from an undetermined site (*n* = 15, 3%). Microbial pathogens were identified in 12 patients with bronchitis (including 5 influenza virus) and 25 with pneumonia (including 11 *Streptococcus pneumoniae*).

The pathophysiological pattern of post-infectious MI was primarily Type 2 (T2MI: 72% of post-infectious MI vs. 13% of other MI, *p* < 0.001). However, the distribution of post-infectious MI varied according to age (Figure 1): with increasing age, the rate of Type 1 post-infectious MI remained stable at roughly 3% of all MI, whereas Type 2 post-infectious MI gradually increased with age, reaching 13% in MI among the >75 y age group. After adjustment for age and sex, acute pneumonia was associated with a 18-fold risk of Type 2 MI vs. Type 1 MI (OR (95% CI) = 18 (12–28)). This risk was even higher after acute pneumonia (OR (95% CI) = 24 (15–38)).

### 3.2. Angiographic Data

As expected, coronary angiography was done less often for post-infectious MI patients than for other patients (78% vs. 97% *p* < 0.001) and more frequently found non-obstructive arteries (14% vs. 6% for other MI, *p* < 0.001) or diffuse CAD (3-vessel disease: 36% vs. 30%, *p* = 0.02). Patients with non-obstructive CAD presented similar characteristics compared with patients with obstructive CAD (Ischemic chest pain in 66% vs. 55% of patients, *p* = 0.2), except for less frequent ST segment elevation (26% vs. 39%, *p* = 0.02). Although CAD severity, assessed with the SYNTAX score, was similar for both groups, percutaneous coronary intervention was much less frequent in the post-infectious MI group (41 vs. 73%, *p* < 0.001).

### 3.3. Hospital Outcomes

**Figure 5:**
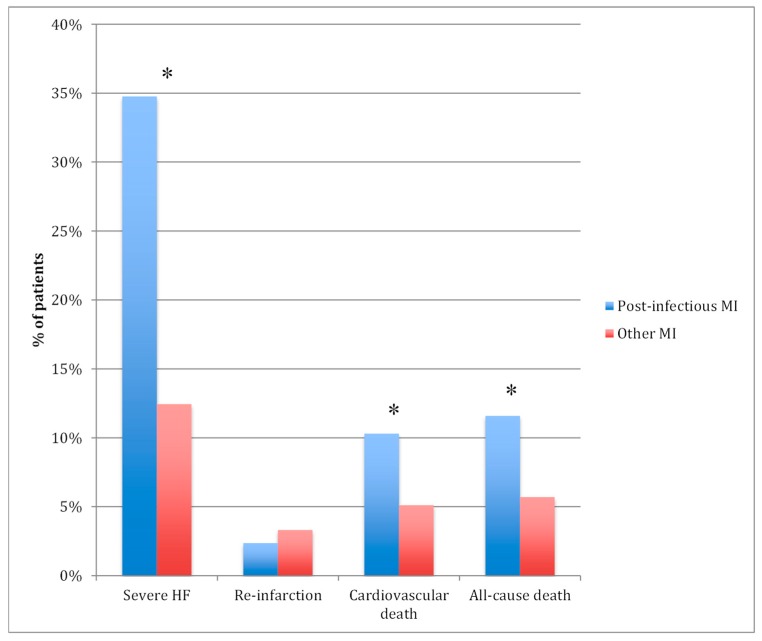
In-hospital outcomes. * *p* < 0.05; HF: heart failure; MI: myocardial infarction.

All-cause in-hospital deaths (Figure 5) were twice as common in post-infectious MI patients (11% vs. 6%, *p* < 0.001), mainly from cardiovascular cause (10% vs. 5%, *p* < 0.001). Among the patients who died in-hospital, the proportion of post-infectious MI gradually increased with age, reaching 23% after 75 years (Figure 1). Severe acute heart failure was three times more common in the post-infectious MI group (35 vs. 12%, *p* < 0.001). However, after adjustment for GRACE risk score and other prognostic factors (Type 1 or 2 MI, LVEF, troponin rate), post-infectious MI was no more associated with worse in-hospital outcome compared with other MI (Table 3) (OR (95% CI) = 0.72 (0.43–1.20) for all-cause mortality and OR (95% CI) = 1.22 (0.85–1.76) for severe heart failure). In-hospital re-infarction was as rare in both groups. There was no significant difference in outcomes between Type 1 and Type 2 post-infectious MI (all cause death at 11% in both groups), except severe acute heart failure which was almost twice more frequent in Type 2 post-infectious MI (40% vs. 23%, *p* < 0.001).

In patients with post-infectious MI, factors associated with risk of in-hospital mortality are presented in Table 4. After adjustment for GRACE risk score and main MI prognosis factors (i.e., CRP, NT-ProBNP levels, LVEF), respiratory tract infection was associated with a threefold increased risk of hospital mortality when compared with other site infections (OR (95%CI) = 2.89 (1.19–6.99), *p* = 0.02). When compared with Type 2 MI, Type 1 MI was also associated with a higher risk of hospital mortality (OR (95%CI) = 2.44 (1.12–5.29), *p* = 0.02). In the subgroup of patients with angiographic data (*n* = 365), this association was even greater after adjustment for CAD severity according to the SYNTAX score (OR (95%CI) = 4.53 (1.64–12.55), *p* = 0.004).

## 4. Discussion

Acute infection and MI are both leading causes of hospitalization and of short-term mortality worldwide. Greater awareness of the association between these two conditions is necessary for clinicians to be able to identify larger numbers of high-risk patients. Even though causal relations between acute infection and MI have already been addressed in one retrospective study [1], this prospective contemporary study is, to the best of our knowledge, the first to individualize post-infectious MI as an individual entity in a large cohort of hospitalized MI and to comprehensively describe the characteristics and outcomes of post-infectious MI. The key results are as follows: (a) a high rate (10%) of post-infectious MI among all hospitalized MI, and gradually increasing with age; (b) a predominance of the Type 2 pathophysiological pattern, especially in older patients (c) a twofold increased risk of in-hospital mortality compared with other MI due to cardiovascular disease severity, as acute infection was no more associated with mortality after adjustment for cardiovascular confounders; (d) respiratory tract infection and T1MI, as situations at highest risk of mortality.

### 4.1. Type of Infection and MI

The relationship between respiratory tract infection and acute MI is no longer debated. Up to half of all excess deaths during flu season could be due to cardiovascular causes [24] and occurrence of MI may be 30% more likely [25]. Acute pneumonia has also been associated with a four-fold increase in risk of cardiovascular events within the first month of admission [14] and with a greater than 100-fold increase in risk in the first 3 days after admission [26]. Incidence of acute MI is 5 times higher among patients hospitalized for pneumonia, when compared with other inpatients [27]. Pneumonia, called the “friend of the aged” by Sir William Osler, remains one of the most deadly diseases in frail comorbid patients, as the trigger of organ failure leading to death, more than as the cause of death itself [28]. Nearly 10% of patients hospitalized for pneumonia presented concurrent MI at admission [6,13,15,26]. These results are consistent with ours, which pointed respiratory tract infection as the leading cause (67%) of post-infectious MI, far ahead of other infections. Bacteremia [29], urinary tract infection [12] and gastroenteritis [1] are also found in acute MI, although with a lower prevalence.

### 4.2. Prognosis of Respiratory Tract Infection in MI

Interestingly, our results showed not only a more frequent association, but also a worse prognosis for cases of post-infectious MI after respiratory tract infections, when compared with other infections. This could partly be explained by the sepsis-related mortality of acute pneumonia, as pneumonia is per se associated with a worse prognosis than urinary tract infection. However, mortality was mostly due to cardiovascular events, supporting the hypothesis that respiratory tract infections may have more cardiovascular consequences than other acute infections. Hypoxemia and systemic stress are factors that potentially favor the risk of myocardial ischemia [30]. However, patients with pneumonia have an increased risk of MI, even after apparent recovery [14]. The activation and aggregation of platelets by bacteria and their byproducts could be involved in the heightened risk of ischemic events [31]. A pro-thrombotic state was observed in pneumonia, with a correlation between endotoxins rate and clotting activation [32], and markers of increased platelet activity have been associated with MI in patients with acute pneumonia [6]. Antiplatelet therapy has been associated with a lower mortality in a large prospective study of elderly patients with acute pneumonia [33] and in one small randomized trial of 185 patients including patients with pneumonia and cardiovascular risk factors [34]. However, this protective effect of low dose aspirin was not confirmed in another observational study focusing on MI occurrence after pneumonia [6]. Pneumonia pathogens may also have a more direct cytotoxic effect on cardiomyocytes, especially in pneumococcal pneumonia. Pneumolysin has indeed been shown to be responsible for cardiac microlesions in experimental animal models and in post mortem studies [31].

One of the main interests of our work was to establish the pathophysiological pattern of post-infectious MI according to the 3rd universal definition of MI [17], recently confirmed by the 4th universal definition [7]. Most post-infectious MI were categorized as Type 2 MI, especially in the older age groups. As post-infectious MI included patients who were 10 years older than patients with other MI, high proportion of Type 2 MI could in part be explained by this older age. Compared with Type 1 MI, Type 2 MI is preferentially found in older frail patients [35]. T2MI pathogenesis is likely multifactorial and frequently associated with additional predisposing factors, more common in older comorbid patients, such as severe anemia, unbalanced by acute triggers [36], especially acute infections, which are also markedly increased with age [37]. Acute infections should not be seen as a unique etiological factor of post-infectious MI genesis, but as one component among other known and unknown contributing factors, which result in acute myocardial mismatch in oxygen supply/demand. Conversely, rate of Type 1 post-infectious MI, a minority in our series (28%), remained stable whatever the age. It results from an angiography-proven acute coronary event, such as plaque rupture, disruption or erosion, in a context of predisposing acute infection-related oxygen imbalance situation. The respective weight of each pathogenesis pattern is unknown and probably highly variable. In an angiography-based case-crossover study, Ruane et al. recently found upper respiratory tract infections to be associated with a higher relative risk of MI. Interestingly, when compared with patients who did not have respiratory tract infection, the increased risk was the highest among patients with incomplete coronary stenosis in angiography (TIMI 2–3) and lower among patients with coronary occlusion (TIMI 0–1), confirming that plaque rupture and thrombosis leading to T1MI may be less frequent in post-infectious MI pathogenesis [38]. However, the angiographic documentation of an acute plaque event associated with acute infection, defining Type 1 post-infectious MI, was linked to a markedly higher risk of hospital mortality, suggesting the marked prognostic impact of coronary plaque events in post-infectious MI. Currently, there is no published data to confirm our results, and further studies are clearly needed to better understand post-infectious MI pathogenesis. In a large retrospective series, Smilowitz et al. demonstrated that patients with sepsis and MI, for whom percutaneous coronary intervention was performed, had lower mortality than patients managed conservatively [39], indicating that coronary revascularization may be beneficial for these patients. However, angiographic findings were unfortunately not available to identify the occurrence of acute plaque events.

Another interesting result is the difference on biomarkers profile in post-infectious MI compared with other MI. Indeed, post-infectious MI was characterized by a high NT-pro Brain Natriuretic Peptide and a low troponin I peak level. Low troponin peak and high elevation of NT-pro Brain Natriuretic Peptide have already been described in Type 2 MI [8,40], predominant in post-infectious MI. Moreover, infection per se has been highlighted as a cause of NT-pro Brain Natriuretic Peptide elevation [41].

### 4.3. Limitations

Several limitations have to be acknowledged. First, this study was limited to patients hospitalized in coronary care units, though post-infectious MI frequently occurs in frail comorbid individuals hospitalized in various departments. This inclusion bias could be responsible for an overestimation of typical MI presentation and an underrepresentation of older, frailer patients. Admission for acute MI outside the cardiology department trends to occur in population which is 10 years older and with more frequent comorbidities, markedly more severe presentation and worse outcomes than patients admitted to cardiology departments [42]. Type 2 MI is also less frequent in cardiology departments [42] and could thus have been underestimated in this study. Secondly, even if coronary angiography was performed in a large majority of patients, we cannot exclude acute coronary plaque events among those without angiography data. In addition, angiography has a weak sensibility for small or eccentric thrombus. Coronary plaque rupture and ulceration could thus have been under-diagnosed and several T1MI may have been misclassified. Third, limited data concerning acute infection characteristics, timing and sepsis severity were collected. However, post-infectious MI mortality was largely from cardiovascular causes and acute infection per se appears to be weakly causal in mortality. Flu vaccination status was not available. Further prospective studies are needed to address the benefice of flu vaccination on post infection MI. Fourth, even using a restrictive definition of acute infection, i.e., combining evocative clinical symptoms and inflammation criteria, the diagnosis of infection is difficult in the acute MI setting, since MI itself triggers an intense inflammatory response [43]. Fifth, patients with post-infectious MI were much older, with more comorbidities and thus were more prompt to develop infection. However, only patients for with acute infection symptoms precede MI symptoms were included. We cannot exclude that in rare cases, acute infection could be a complication, and not a cause, of cardiovascular event. Indeed, heart failure in acute MI has been associated with an almost doubled risk of pneumonia in epidemiological studies [44,45]. In congestive heart failure, the pulmonary alveolar flooding may result in increasing the risk of infection and hampering clearance once infection is established [46]. Finally, the fourth universal definition has newly distinguished Type 2 MI from myocardial injury, also frequently associated with acute infection. However, as only patients with clinical, imaging or ECG signs of myocardial ischemia have been included, which is consistent with the new definition, non-ischemic myocardial injury have thus implicitly not been included in this study.

## 5. Conclusions

In this large study from a regional prospective survey, acute infection was common (10% of admissions for MI). For a vast proportion, it concerned respiratory tract infection-triggered MI in an elderly comorbid population. Considering a causal link has already been established between acute infection and MI, our findings strongly suggest a new nosological entity: post-infectious MI. Mortality was two times higher than other MI, mostly due to cardiovascular events, and respiratory tract infection was an independent predictor of worse prognosis. Although acute plaque events (Type 1 MI) were found by angiography in less than 30% of patients, suggesting Type 2 MI to be far more common in post-infectious MI pathogenesis, they were associated with a worse in-hospital prognosis. Further studies are needed to establish if early recognition of acute infection as a curable trigger in case of MI, and early detection of post-infectious MI after respiratory tract infection, could both improve prognosis. Identification of the patients at highest risk of post-infectious MI could contribute to target future specific therapeutic recommendations and prevention strategies such as pneumococcal/flu vaccination and intensive cardiovascular preventive management.

## Figures and Tables

**Table:** 

	AUC (95% CI)	Cutoff	Sensitivity	Specificity	*p-*Value
**C-reactive protein**	0.75 (0.72–0.78)	8.6 mg/L	73%	67%	<0.001
**NT- pro BNP**	0.72 (0.69–0.75)	1944 pg/mL	65%	68%	<0.001
**Troponin I**	0.43 (0.40–0.46)	12 µg/L*	50%*	62%*	<0.001

* for prediction of non post-infectious MI. AUC: area under the curve; CI: confidence interval; NT-pro BNP: N-terminal- pro brain natriuretic peptide.

**Table 1:** Patients characteristics on admission (*n* (%) or median (IQR)).

	Post-Infectious MI *n* = 466	Other MI *n* = 4107	*p*-Value
**Risk factors and comorbidities**			
Age, years	78 (66–85)	68 (57–80)	<0.001
Female	188 (40)	1166 (28)	<0.001
BMI, kg/m²	26 (23–30)	26 (24–30)	0.07
Hypertension	343 (74)	2454 (60)	<0.001
Hypercholesterolemia	249 (53)	2144 (52)	0.4
Family history of CAD	134 (28)	1330 (32)	0.03
Smoking	78 (17)	1232 (30)	<0.001
Diabetes	186 (40)	1026 (25)	<0.001
Chronic renal failure	66 (14)	217 (5)	<0.001
COPD	81 (17)	291 (7)	<0.001
Neoplasia	90 (19)	565 (14)	0.003
**Cardiovascular history**			
CAD	168 (36)	985 (24)	<0.001
Stroke	56 (12)	315 (8)	0.003
PAD	84 (18)	316 (8)	<0.001
HF	61 (13)	187 (5)	<0.001
Atrial fibrillation	100 (21)	400 (10)	<0.001
Aortic stenosis	50 (11)	189 (4)	<0.001
**Type of MI**			
Type 1	130 (28)	3580 (87)	<0.001
Type 2	336 (72)	527 (13)	<0.001
**Clinical data at admission**			
HR, beats/min	83 (70–100)	78 (67–90)	<0.001
SBP, mmHg	131 (114–154)	140 (120–160)	<0.001
DBP, mmHg	72 (62–87)	80 (70–93)	<0.001
Anterior wall location	179 (38)	1457 (35)	0.2
GRACE risk score	177 (151–199)	144 (121–173)	<0.001
Acute HF	252 (54)	957 (23)	<0.001
LVEF, %	45 (35–55)	55 (45–60)	<0.001
**ECG at admission**			
STEMI	181 (39)	1969 (48)	<0.001
AF/Flutter	64 (14)	362 (9)	<0.001
LBBB	48 (10)	233 (6)	<0.001
**Biological data**			
Hemoglobin, g/100mL	12.8 (11.6-14.3)	14.2(12.9–15.3)	<0.001
Leucocytes, G/L	12.6 (9.9-14.7)	12.0 (9.7–14.1)	<0.001
CRP, mg/L	33 (7–103)	5 (3–13)	<0.001
Creatinine, µmol/L	93 (70–130)	83 (70–104)	0.003
eGFR, mL/min	60 (39–84)	77 (57–93)	<0.001
Troponin I peak, µg/L	7 (2–29)	13 (3–58)	<0.001
NT-proBNP, pg/mL	3800 (920–12772)	664 (164–2685)	<0.001
CK peak, IU/L	340 (131–991)	496 (189–1457)	<0.001
**Angiographic data**			
Coronary angiography	365 (78)	4006 (97)	<0.001
Non-obstructive/normal	53 (14)	246 (6)	0.005
3-vessel disease	132 (36)	1216 (30)	0.02
SYNTAX score	11 (3–20)	10 [5,6,7,8,9,10,11,12,13,14,15,16,17,18]	0.8
**Acute management**			
PCI	190 (41)	2994 (73)	<0.001
CABG	193 (5)	18 (4)	0.4
**Hospital outcomes**			
ICU stay (days)	4 (3–6)	4 (3–5)	<0.001
Hospital stay (days)	13 (7–21)	9 (7–12)	<0.001
All cause death	54 (11)	234 (6)	<0.001
Cardiovascular death	48 (10)	210 (5)	<0.001
Re-infarction	11 (2)	136 (3)	0.3
Severe HF	162 (35)	511 (12)	<0.001

AF: atrial fibrillation; BMI: body mass index; CABG: coronary artery bypass grafting, CAD: coronary artery disease; CK: creatine kinase; COPD: chronic obstructive pulmonary disease; CRP: C-reactive protein; DBP: diastolic blood pressure; eGFR: estimated glomerular filtration rate; HF: heart failure; HR: heart rate; ICU: intensive care unit; IQR: interquartile range; LBBB: Left bundle branch block; LVEF: left ventricular ejection fraction; MI: myocardial infarction; NT-proBNP: N-Terminal pro Brain Natriuretic Peptide; PAD: peripheral arterial disease; PCI: percutaneous coronary intervention; SBP: systolic blood pressure; STEMI: ST segment elevation myocardial infarction.

**Table 2:** Characteristics of post-infectious myocardial infarction at admission (*n* (%)).

MI Characteristics	
Ischemic chest pain	256 (55)
New ECG abnormalities	384 (82)
ST segment elevation	181 (39)
ST segment depression	121 (26)
T wave inversion	112 (24)
New LBBB	21 (5)
New pathological Q waves	111 (24)
Imaging evidence of ischemia	59 (13)
**Infection characteristics**	
Temperature >39°C	134 (29)
Respiratory rate > 24/min	153 (33)
Leucocytes > 12 × 10^9^/L	151 (32)
Heart rate > 100/min	114 (24)
Respiratory tract infection	313 (67)
Acute bronchitis	163 (35)
With microbial identification:	12
*Influenzae virus*	5
*Parainfluenzae virus*	2
*Metapneumovirus*	1
*Rhinovirus*	2
*RSV*	2
Acute pneumonia	150 (32)
With microbial identification:	25
*Streptococcus pneumoniae*	11
Other *Streptococcus spp.*	3
*Enterococcus spp.*	2
*Haemophilus influenzae*	2
*Escherichia coli*	2
*Pseudomonas aeruginosa*	1
*Citrobacter koseri*	1
*Hafnia alvei*	1
*Moraxella catarrhalis*	1
*Aspergillus fulmigatus*	1
Urinary tract infection	78 (17)
Other site infection	60 (13)
Undetermined infection	15 (3)

LBBB: Left Bundle Branch Block; MI: Myocardial Infarction; RSV: Respiratory Syncytial Virus.

**Table 3:** Logistic regression analysis of factors associated with main in-hospital outcomes after MI (*n* = 4573).

	Severe HF (*n* = 673)			CV Mortality (*n* = 258)			All-Cause Mortality (*n* = 288)		
	OR	95% CI	*p*-Value	OR	95% CI	*p*-Value	OR	95% CI	*p*-Value
Post-infectious MI (*vs. other MI*)	1.22	0.85–1.76	0.3	0.87	0.50–1.50	0.6	0.72	0.43–1.20	0.2
Type 1 MI *(vs. Type 2)*	**0.41**	**0.30–0.55**	**<0.001**	0.69	0.44–1.08	0.1	**0.53**	**0.35–0.80**	**0.003**
GRACE Score *(per point)*	**1.04**	**1.03–1.04**	**<0.001**	**1.03**	**1.03–1.04**	**<0.001**	**1.03**	**1.03–1.04**	**<0.001**
LVEF *(per 10 %)*	**0.61**	**0.56–0.67**	**<0.001**	**0.66**	**0.58–0.75**	**<0.001**	**0.68**	**0.60–0.76**	**<0.001**
Troponin (per 10 *µg/L*)	**1.01**	**1.00–1.02**	**<0.001**	**1.02**	**1.01–1.03**	**<0.001**	**1.02**	**1.01–1.03**	**<0.001**

CI: confidence interval CRP: C reactive protein; LVEF: left ventricular ejection fraction; MI: myocardial infarction; NT-proBNP: N-terminal pro brain natriuretic peptide; OR: odds ratio; RTI: respiratory tract infection.

**Table 4:** Logistic regression analysis of factors associated with in-hospital mortality after post-infectious myocardial infarction (*n* = 466).

	Univariable			Multivariable		
	OR	95% CI	*p*-Value	OR	95% CI	*p*-Value
GRACE Score *(per point)*	**1.02**	**1.01–1.03**	**<0.001**	**1.02**	**1.01–1.03**	**0.002**
Type 1 MI (vs. *Type 2*)	0.98	0.53–1.87	1	**2.44**	**1.12–5.29**	**0.02**
RTI (vs. *other infection*)	1.46	0.77–2.76	0.3	**2.89**	**1.19–6.99**	**0.02**
LVEF *(per 10 %)*	**0.66**	**0.52–0.83**	**<0.001**	**0.74**	**0.55–0.99**	**0.04**
CRP *(per 10 mg/L)*	**1.06**	**1.03–1.09**	**<0.001**	**1.05**	**1.02–1.09**	**0.005**
NT-proBNP *(per 1000 pg/mL)*	**1.03**	**1.01–1.04**	**0.006**	**1.02**	**1.01–1.03**	**0.007**

CI: confidence interval CRP: C-reactive protein; LVEF: left ventricular ejection fraction; MI: myocardial infarction; NT-proBNP: N-terminal pro brain natriuretic peptide; OR: odds ratio; RTI: respiratory tract infection.
